# Engineering broad-spectrum resistance to cotton leaf curl disease by CRISPR-Cas9 based multiplex editing in plants

**DOI:** 10.1080/21645698.2021.1938488

**Published:** 2021-06-14

**Authors:** Muhammad Salman Mubarik, Xiukang Wang, Sultan Habibullah Khan, Aftab Ahmad, Zulqurnain Khan, Muhammad Waqas Amjid, Muhammad Khuram Razzaq, Zulfiqar Ali, Muhammad Tehseen Azhar

**Affiliations:** a Centre of Agricultural Biochemistry and Biotechnology (CABB), University of Agriculture, Faisalabad, Pakistan; b College of Life Sciences, Yan’an University, Yan’an, Shaanxi, China; c Department of Biochemistry, University of Agriculture, Faisalabad, Pakistan; d Institute of Plant Breeding and Biotechnology (IPBB), MNS University of Agriculture, Multan, Pakistan; e State Key Laboratory of Crop Genetics and Germplasm Enhancement, Nanjing Agricultural University, Nanjing, China; f Soybean Research Institute, National Center for Soybean Improvement, Nanjing Agricultural University, Nanjing, China; g Institute of Molecular Biology and Biotechnology, Bahauddin Zakariya University, Multan, Pakistan; h School of Agriculture Sciences Zhengzhou University, Zhengzhou, China

**Keywords:** Geminiviruses, virus interference, multiplex editing, broad-spectrum resistance, food security

## Abstract

Advances in genome editing technologies have tremendous potential to address the limitations of classical resistance breeding. CRISPR-Cas9 based gene editing has been applied successfully in plants to tolerate virus infections. In this study, we successfully tested CRISPR-Cas9 system to counteract cotton leaf curl disease (CLCuD) caused by whitefly transmitted cotton leaf curl viruses (CLCuVs). We also analyzed the ability of CLCuV to escape the Cas9 endonuclease activity. Targeting overlapping genes of most prevalent CLCuVs with three gRNAs resulted in virus interference, as validated by low virus titer. Furthermore, multiplex CRISPR-Cas9 construct simultaneously targeting six genes of CLCuV, was found more effective to interfere with virus proliferation compared to targeting single region individually. Additionally, transgenic *N. benthamiana* plants expressing multiple gRNAs simultaneously showed enhanced tolerance against CLCuV infection when compared to wild-type plants. T7 Endonuclease-I (T7EI) assay, showing indels in the CLCuV genome, confirmed the occurrence of double strand breaks (DSBs) in DNA at target sequence induced by Cas9 endonuclease. We observed that targeting CLCuV genome at multiple sites simultaneously resulted in better interference, also with inefficient recovery of altered virus molecules. Next, we tested multiplex construct in cotton to interfere CLCuV infection. We found significant decrease in virus accumulation in cotton leaves co-infiltrated with multiplex cassette and virus compared to cotton leaves infiltrated with virus only. The results demonstrate future use of CRISPR-Cas9 system for engineering virus resistance in crops. Moreover, our results also advocate that resistance to mixed virus infections can be engineered using multiplex genome editing.

## Introduction

Begomoviruses transmitted viral diseases threaten crop production worldwide. The situation has deteriorated in underdeveloped countries, especially in areas where crop-free seasons are unusual and plants are constantly exposed to vector-borne viral diseases.^1^ Losses due to whitefly-transmitted begomoviruses in agriculture presents a huge challenge for researchers in relation to yield and quality of crop plants.^2^ Begomoviruses infection causes leaf curling, yellow discoloration and stunted growth leading to reduced yield.^3^ It is difficult to find a field without virus infection over several kilometers once whiteflies transfer begomoviruses into plants. Based on genome arrangement and phylogenetic studies, begomoviruses considered as highly dynamic group of viruses. Such viruses are classified into old world and the new world viruses.^4,5^ The new world begomoviruses are bipartite while the old world includes both bipartite and monopartite. The bipartite genome has two main components known as DNA-A and DNA-B, and an extra bipartite-like monopartite genome identical to DNA-A. Usually, DNA-A possesses six open reading frames (ORFs), while DNA-B includes two ORFs encoding movement protein and nuclear shuttle protein.

Genome engineering has been on the forefronts to produce high yielding climate resilient crops.^6^ However, still there is yawning gap to combat challenges. Biologists are now more equipped to manipulate genes to enhance yield and resistance of crop varieties under different biotic and abiotic stresses.^7^ Advance genome engineering techniques offer maneuvering of the genome in precise manner i.e. editing, deleting or addition of genes site specifically.^8^ These genome modifications are more feasible alternative as compared to conventional methods of transgenic development.^9^ The ability to induce site-specific DSBs at the position of interest should be taken as rear-guard action before utilizing it at full scale. Recently, precise genome engineering approaches such as Zinc Finger Nucleases (ZFNs), Transcription Activators Like Effector Nucleases (TALENs) and Clustered Regularly Interspaced Short Palindromic Repeats-CRISPR associated-9 (CRISPR-Cas9) have been shown to induce favorable genetic alterations.^10^ The CRISPR-Cas9 system is a natural immune system in bacteria and archaea against invasion of viral or plasmid DNAs.^11^ It has been considered as a promising and versatile tool to reshape the genome engineering dimensions in agriculture.^12^ This approach is anticipated to result a broad range of applications in genome engineering experiments involving crop plants and devising multigenic disease resistance models. The programmable CRISPR-Cas9 system is a robust and inexpensive approach with great potential to bring a next-generation of gene editing experiments.

Since conventional breeding approaches have not been much successful against begomoviruses infection, due to multiple virus strains and complex interaction of host plant, whitefly vector and virus particles. However, potential use of CRISPR-Cas9 based virus genome editing can be effective to improve agricultural productivity.^13^ Initial reports of engineering plants with RNA-guided Cas9 targeting the highly destructive Geminiviruses have been shown successful virus genome interference.^14–18^ Other studies have recently demonstrated the deployment of the CRISPR-Cas9 system to target coding or non-coding viral DNA regions in plants with varying gRNA efficiencies.^19–21^ These studies showed delayed symptoms of the disease and reduced the virus accumulation in plants treated with CRISPR-Cas9 reagents. However, some reports described the emergence of virus variants capable of replicating and with different level of disease infections.^19,22^

Previously, several gRNAs were individually screened to thwart virus disease symptoms, limited or no efforts are reported of targeting plant virus genome at multiple sites with more than one gRNAs simultaneously. Developing durable and broad-spectrum tolerance under open-field conditions is a serious challenge because of mixed viral infections.^23^ Multiple gRNAs should be required to target key DNA sequences at same time. Multiplexing potential gRNAs should be used to test the additive effects of multiple gRNAs on reduced virus accumulation and severity of the disease symptoms.^19,22,23^ In the present research, we initially analyzed the ability of the CRSIPR-Cas9 system to interfere individually with overlapping genes of three most prevalent CLCuVs in Pakistan; cotton leaf curl curl Multan virus (CLCuMuV), cotton leaf curl Burewala virus (CLCuBuV), and cotton leaf curl Kokhran virus (CLCuKoV). We found that three individual gRNAs designed to target AV2/AV1, AC2/AC3 and AC1/AC4 overlapping gene sequences targeted the respective sites in three CLCuV strains. Furthermore, we predicted that simultaneous targeting of six CLCuV genes would be a better choice to tackle virus proliferation inside the plants. We examined the ability of three gRNAs in a single construct to inhibit CLCuV replication in plants. Our results indicated that multiplexed CRISPR-Cas9 approach provided a highly efficient virus genome interference and reliable approach for developing broad spectrum and durable plant tolerance against begomoviruses.

## Material and Methods

### Designing of gRNAs in Overlapping CLCuV Genes

We utilized the three CLCuV strains for DNA sequences analysis using CLUSTAL-W and T-Coffee online web tools. Consensus sequences in overlapping genes were selected to design potential gRNA targets using CRISPR-Multitargeter software, http://www.multicrispr.net/multalign_input.html. Three potential and unique gRNA targets were found, present in consensus sequences of six overlapping genes of CLCuV DNA-A. These targets were also compared with CRISPR-P 2.0 recommended gRNA targets to validate the accuracy of CRISPR-Multitargeter software.

### Plasmids and Cloning of gRNAs in Plant Expression Vector

The pHSE401 plant expression vector (Addgene plasmid # 622010) was used for cloning and expression of both gRNA and Cas9 in plants. Two *Bsa*I restriction sites in the pHSE401 vector were used for individual gDNA cloning. All gDNA oligos containing *Bsa*I adapter sequences at the 5´ends were commercially synthesized for cloning. The pHSE401 was digested with *Bsa*I, treated with alkaline phosphatase, and purified with a GeneJET gel extraction kit (Thermo Fisher Scientific, Cat. No. K0502). Individual oligos (Tab. 1) were first annealed by combining 1 μL of each oligo (100 μM) with 1X T4 DNA ligase buffer in a total reaction volume of 10 μL. The reaction was heated to 95 °C for 5 min and then left at room temperature for 30 min. These annealed gDNA oligo pairs were ligated into *Bsa*I-digested pHSE401 using T4 DNA ligase (Thermo Fisher Scientific, Cat. No. EL0011). Positive clones were confirmed by sequencing using M13-F primers (Tab. 1). The multiplexed CRISPR-Cas9 vector was commercially synthesized from VectorBuilder, https://en.vectorbuilder.com/.

**Table 1.** List of primers used in this study

Primer	5ʹ to 3ʹ Sequence	Function
V2V1-gRNA	ATTGTTTGGTGACGCGGACAATGG	gRNA cloning
	AAACCCATTGTCCGCGTCACCAAA	
C2C3-gRNA	ATTGCTTAAGAAACGACCAGTCGG	gRNA cloning
	AAACCCGACTGGTCGTTTCTTAAG	
C1C4-gRNA	ATTGTACGCCGCAGCACTTAACGC	gRNA cloning
	AAACGCGTTAAGTGCAGCGGCGTA	
M13 Forward	TGTAAAACGACGGCCAGT	Confirmation of gRNA integration
CLCuV-V2V1	TCGGCCAATCATATGACGCG	Indel detection
	CATACTTGCCAGCTTCCTGC	
CLCuV-C2C3	GCAGGAAGCTGGCAAGTATG	Indel detection
	CGTCATTGATGACGTCGACC	
CLCuV-C1C4	TTCTTGGCTAGCCTGTGCTG	Indel detection
	ACGAAGATGGGACTCCTCAC	

### Plant Growth and Transient Transformation

Few seeds of *N. benthamiana* and cotton were grown in small pots containing peat moss and soil at ambient temperature with 8 h dark and 16 h light photoperiod [80–100 μmol/m^2^s^−1^). When plants achieved the optimum stage of development marked by 3–4 fully developed true leaves with no clear flower buds were used for infiltration studies. Transient expression of all constructs was performed by agroinfiltration according to the method described by.^24^

### Disease Resistance Assay

For disease resistance assay, infectious clones of CLCuKoV, cotton leaf curl Multan beta-satellite (CLCuMuB] and CRISPR-Cas9 constructs were infiltrated in the *N. benthamiana* and cotton leaves. Infectious clones mentioned throughout the text included DNA-A of CLCuKoV strain and beta-satellite of CLCuMuB. Agrobacterium transformed single colony of each constructs were grown overnight at 37ᵒC with 180 rpm, centrifuged, and resuspended in infiltration solution (Acetosyringone 150uM, 10 mM MgCl_2_ and 10 mM MES pH 5.8). Resuspended agrobacterium cultures were incubated in the dark for 2–4 hours at ambient temperature. Bacterial cultures were infiltrated by 1 ml needleless syringe into 3–4 weeks old leaves of *N. benthamiana* plants and 4–5 weeks old cotton plants.

### Transgenic Plant Development

*Agrobacterium tumefaciens* (EHA105 strain) cells were transformed with the multiplex CRISPR-Cas9 (pHSE401-MV) plasmids by electroporation, and transformed single bacterial colony was used to transform *In vitro* grown *N. benthamiana* leaf discs, as described by UC Davis Plant Transformation Facility Center (Unpublished method). Cas9 gene specific primers were used to check the integration of multiplex cassette. Expression of gRNAs and Cas9 was measured by qPCR, actin gene of *N. benthamiana* was used as an internal control and reaction normalizer.

### Mutation Detection

To validate the activity of the CRISPR-Cas9 constructs on the CLCuV genome, mutation resulting from DNA DSB were verified by the non-homologous end joining [NHEJ) repair pathway, as described by.^16^ Firstly, PCR amplified genomic DNA using primers encompassing the target sites of the gRNAs (Tab. 1). To confirm the DNA DSB, PCR amplicons were gel purified, denatured, reannealed, and treated with T7EI (NEB, Cat. No. M0302S]. The PCR amplicons were sequenced to determine the mutation frequency.

## Results

### Efficiency of CRISPR-Cas9 Constructs in Transient Assays

**Figure 1.**
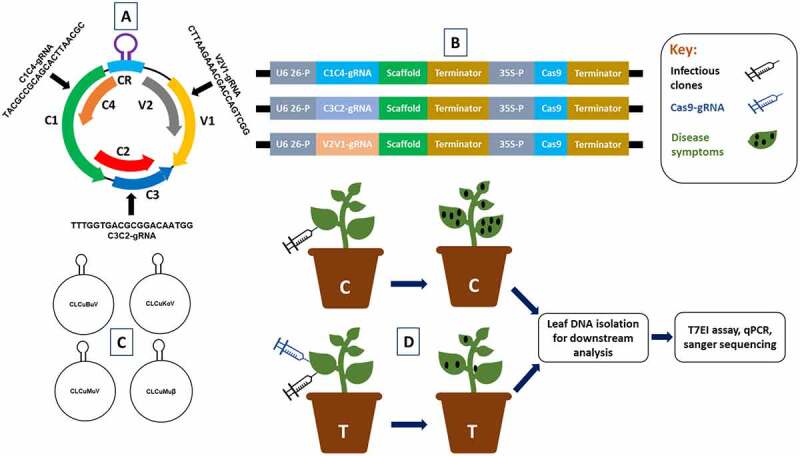
Schematic illustration of CRISPR-Cas9-mediated CLCuV interference in plants. (a) CLCuV DNA-A genome their corresponding genes and three gRNA targets. (b) CRISPR-Cas9 cassettes. (c) Infectious clones of most prevalent CLCuV species. (d) In control plants only CLCuV infectious clones were inoculated, moreover, in experimental treatment plants CLCuV infectious clones followed by Cas9-gRNA cassettes were inoculated. The Cas9-gRNA complex targets the CLCuV DNA at complementary gRNA target sites and induce a DSBs. Downstream analysis showed the degradation of CLCuV DNA.

The CRISPR-Cas9 emerged as an efficient system to interfere various DNA viruses by targeting their essential genomic sequences. We target the six overlapping genes of CLCuV with three individual gRNAs. The annealed gDNA oligos were cloned into a binary vector having plant codon optimized Cas9. To test the efficacy of individual gRNAs to confer virus resistance, agroinfiltration mediated transformation of CRISPR-Cas9 constructs was performed in *N. benthamiana* plants. Next, Cas9-gRNAs infiltrated *N. benthamiana* plants were challenged with the infectious clones of CLCuKoV and helper betasatellite, CLCuMuB for symptoms development (Fig. 1). Based on CLCuD disease symptoms, *N. benthamiana* plants infiltrated with CRISPR reagents and virus infectious clones displayed less and delayed disease symptoms. The virus accumulation analyzed by qPCR showed a decrease in virus titer by 60–70% in systemic leaves of co-infiltrated plants as compared to control plants inoculated with virus alone. Cas9 and distinctly individual gRNA expression validate the low virus titer exhibited by leaves treated with CRISPR-Cas9 constructs.

**Figure 2.**
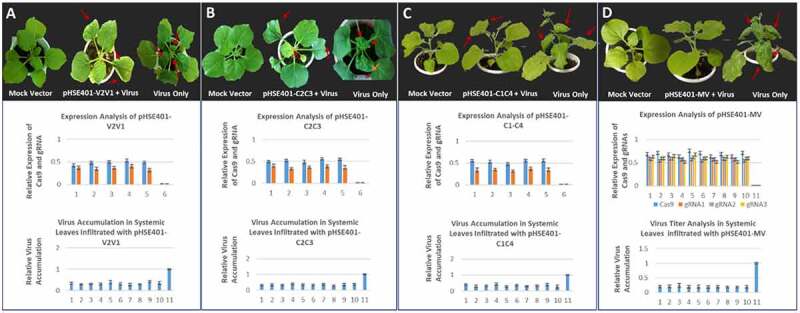
Transient disease resistance assays. Red arrows are indicating CLCuD symptoms. (a) Inoculation of CLCuV infectious clones and Cas9-V2V1-gRNA cassette, expression analysis of Cas9 and V2V1-gRNA and CLCuV accumulation. (b) Inoculation of CLCuV infectious clones and Cas9-C2C3-gRNA cassette, expression analysis of Cas9 and C2C3-gRNA and CLCuV accumulation. (c) Inoculation of CLCuV infectious clones and Cas9-C1C4-gRNA cassette, expression analysis of Cas9 and C1C4-gRNA and CLCuV accumulation. (d) Inoculation of CLCuV infectious clones and Cas9-MV-gRNA cassette, expression analysis of Cas9 and MV-gRNA and CLCuV accumulation.

Recently,,^22^demonstrated that targeting virus genome with single gRNA resulted in the emergence of virus variant resistant to Cas9 activity. Therefore, based on low virus accumulation and less symptom severity, three gRNAs targeting six overlapping genes of CLCuV were selected for the development of multiplex CRISPR-Cas9 vector (pHSE401-MV). Next, we explored the utility of multiplex CRISPR-Cas9 vector to confer resistance against infection caused by CLCuV. The multiplex vector was transformed in *N. benthamiana* leaves through agroinfiltration and infiltrated leaves were challenged with CLCuV infectious clones. Plants co-infiltrated with multiplex construct and virus showed late and very less virus symptoms. The virus titer in systemic leaves analyzed by qPCR analysis showed reduction in virus accumulation by 80% in co-infiltrated plants as compared to plants infiltrated with virus alone. The expression analysis Cas9 and gRNAs of multiplex CRISPR cassette further reinforce the less disease symptoms and low virus titer (Fig. 2).

### Efficiency of Multiplex CRISPR/Cas9 Construct after Stable Integration

**Figure 3.**
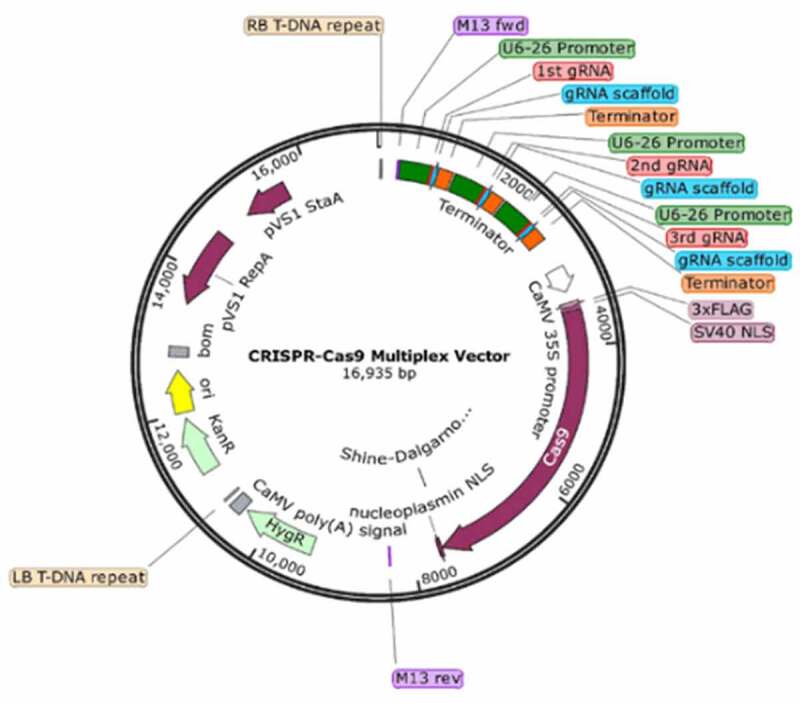
Schematic illustration of multiplex CRISPR-Cas9 vector. A binary CRISPR-Cas9 vector containing three distinct individual gRNAs and Cas9 gene.

We used Agrobacterium mediated leaf disc transformation method to express multiplex CRISPR-Cas9 construct (three distinct gRNAs and Cas9) (Fig. 3). in the *N. benthamiana* plant. The primary putative transformants were selected on culture media with hygromycin (25 mg/L) as the selection marker and transferred to soil for acclimatization and seed harvesting. Five individual lines were selected for transgene screening. DNA was isolated from 5 well-rooted transgenic plants and Cas9 specific primers were used to check the integration of multiplex cassette. The qPCR of selected plants confirmed the expression of both Cas9 and gRNAs in transgenic *N. benthamiana* plants. A slight variation in the expression of Cas9 and gRNAs were observed among selected transgenic lines. Transgenic *N. benthamiana* line expressing highest level of Cas9 and all three gRNAs was selected for virus infectivity assays.

**Figure 4.**
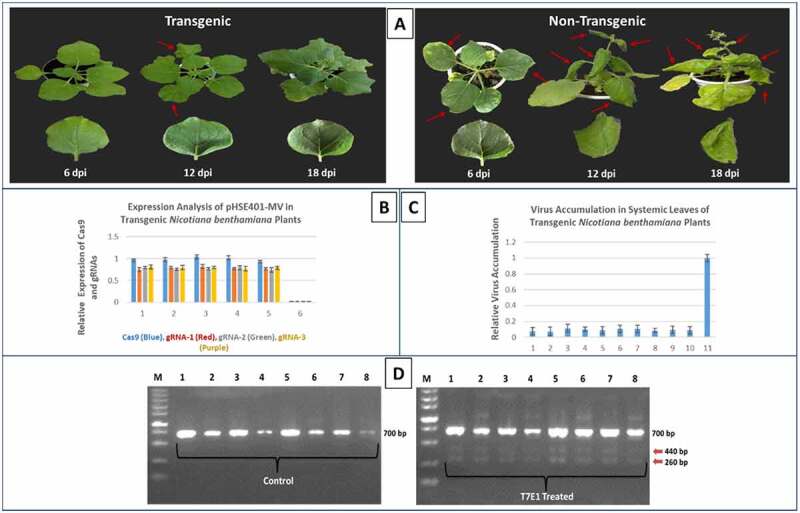
Disease resistance assay in CRISPR-Cas9 transgenic plants. Transgenic plants expressing all three gRNAs and Cas9 were resistance against CLCuV infectious clones and exhibiting no disease symptoms even after 18 dpi (a). It is also validated by analyzing the expression of Cas9 and gRNAs (b), measuring relative virus accumulation (c), and T7EI assay (d). However, non-transgenic plants displaying severe disease symptoms even after 12 dpi (A).

**Figure 5.**
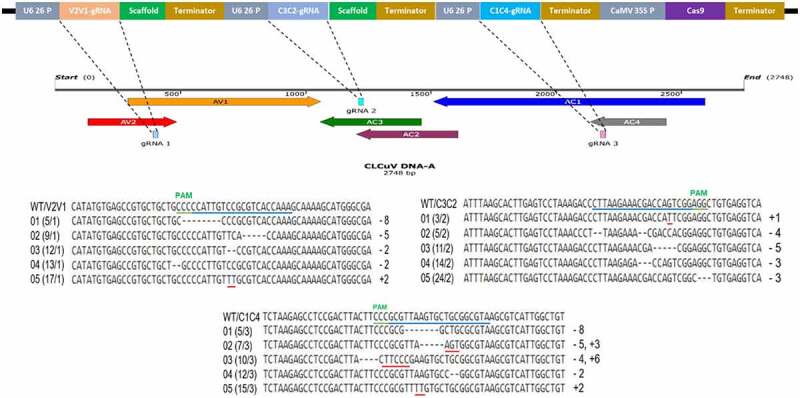
Sequencing of multiplexed CRISPR-Cas9 edited CLCuV DNA in transgenic plants. Alignment of wild-type and CRISPR-edited CLCuV DNA-A sequences encompassing gRNA target sequence displaying different type of indels formation in transgenic plants infiltrated with virus infectious clones at gRNA targeted sites. Blue underline representing 20 bp gRNA sequence and green underline representing PAM region. Red underlines indicating addition of new bases and dots indicating deletion of bases compared with wild type sequence.

To confirm that *N. benthamiana* line expressing multiplex CRISPR-Cas9 cassette was capable of interfering CLCuV genome, the virus infectious clones were infiltrated into the transgenic and wild-type *N. benthamiana* plants. Virus infiltrated transgenic *N. benthamiana* plants displayed less or no disease symptoms compared to non-transgenic plants. A virus titer analysis by qPCR clearly demonstrated very low virus accumulation (up to 95%) in systemic leaves (Fig. 4). Next, we isolated the plant DNA of infiltrated leaves of both transgenic and non-transgenic plants to validate the CRISPR-Cas9 mediated DSBs at targeted sequences. PCR amplicons encompassing the gRNA target sites were denatured, reanneal, and subjected to T7EI for mutation detection analysis. Indel formations confirmed the efficient targeting of overlapping genes of CLCuVs genome in the transgenic plants expressing multiplex CRISPR-Cas9 cassette. Moreover, sequencing results also confirmed Cas9 induced indels formations (Fig. 5). These results clearly demonstrated the high efficiency of simultaneous targeting of the CLCuV genome at multiple sequences enhance plant resistance against virus infection.

For engineered virus resistance, silencing of integrated cassette is common in plants. Therefore, we repeated the virus infectivity assay in T1 and T2 generations to check any variation or evasion of Cas9 endonuclease activity. Consistent with virus infectivity resistance assay in T0 progeny, virus accumulation was remained low and similar results were observed in T1 and T2 generations. Moreover, it was also observed that in addition to less virus accumulation and symptom severity, transgenic plants also showed symptoms recovery between 12–18 dai of virus.

### Fate of Double Strand Breaks in Virus inside Transgenic Plant Cells

**Figure 6.**
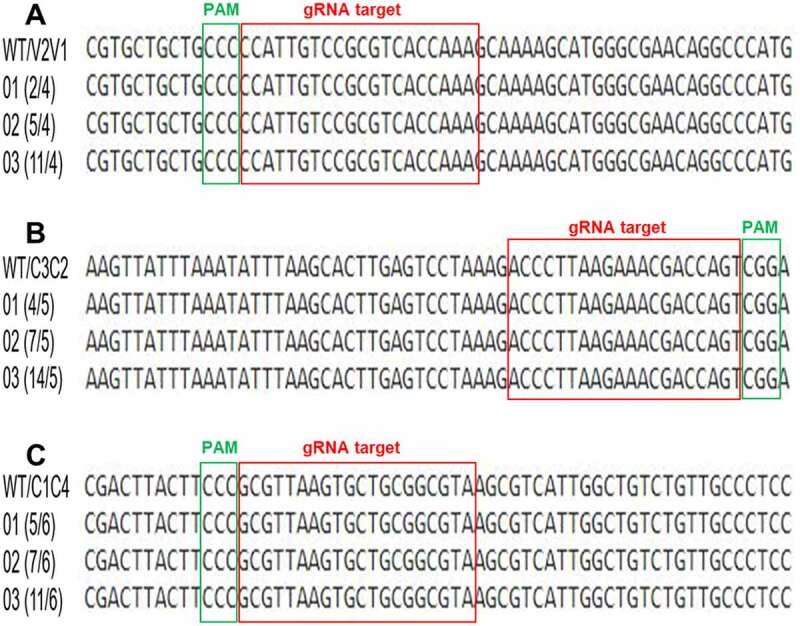
Alignment of CLCuV DNA-A sequences from systemic leaves of transgenic plants. Green box representing PAM region whereas, red box indicating 20 bp gRNA sequence. Sequencing of transgenic plants expressing multiplex cassette inoculated with CLCuV infectious clones exhibiting no indel formation when compared to wild-type sequence.

Virus genome editing provides two possibilities: virus degradation and the non-homologous end joining (NHEJ) pathway for virus repair. Previously, it is reported that virus DNA molecules targeted by CRISPR-Cas9 machinery, capable of replicating and produced disease symptoms.^16,22^ We checked the previously highlighted concern of the evolution of virus variants by targeting with CRISPR-Cas9 machinery to escape resistance. It is anticipated that these virus variants would be un-targetable by Cas9 and freely proliferate across the plants. To test the ability of CLCuV to overcome the multiplex CRISPR-Cas9 construct, we extracted cell sap from the systemic leaves of virus infiltrated transgenic *N. benthamiana* plants expressing multiplex CRISPR-Cas9 cassette and infiltrated into wild-type *N. benthamiana* plants. Total DNA was isolated and used for downstream analysis. When we sequenced PCR amplicons encompassing gRNA target sites, we did not find any change in virus DNA sequence compared to wild type. (Fig. 6). These findings evidently indicated that targeting virus DNA simultaneously at multiple sites resulting into large DNA deletions that are incapable of repair by NHEJ pathway.

### Efficiency of Multiplex Construct in Cotton through Transient Assays

**Figure 7.**
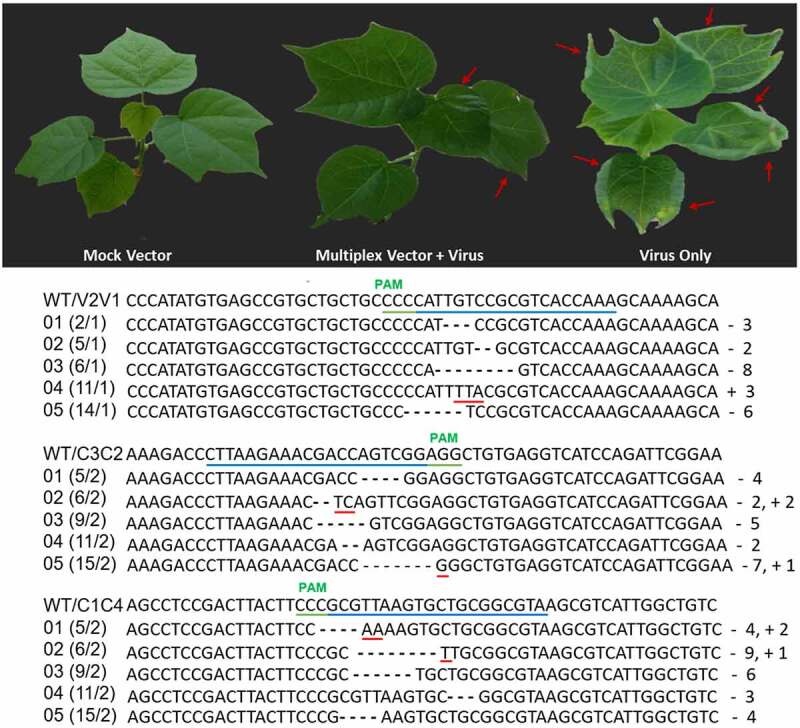
Transient disease resistance assay in cotton plant. Cotton plants inoculating with multiplex CRISPR-Cas9 cassette followed by CLCuV infectious clones displayed tolerance against CLCuV infectious clones and exhibiting very less disease symptoms when compared to cotton plants only inoculated with virus. Red arrows indicating CLCuD symptoms. Sequencing results of multiplex vector inoculated plants exhibited indels formation in CLCuV DNA when compared with wild type sequence.

Cotton leaf curl disease (CLCuD), caused by CLCuV complex, is a major factor affecting cotton production worldwide. Developing resistance cotton cultivars against CLCuD under natural field conditions is a major challenge due to occurrence of multiple CLCuV strains compared to controlled conditions. We first optimized the CLCuV interference in model plant (*N. benthamiana*) and screened highly efficient gRNAs targeting multiple CLCuV strains. Cotton plants were chosen to test the efficacy of multiplex construct against CLCuV infection. Multiplex CRISPR-Cas9 cassette was transformed into cotton plants by agroinfiltration and subsequently challenged with virus infectious clones. We observed virus symptoms on plants infiltrated with virus only and mild symptoms on plants infiltrated with CRISPR-Cas9/virus 10 dai. Virus titer analysis by qPCR showed reduced virus accumulation in multiplex CRISPR-Cas9 infiltrated plants when compared to control plants infiltrated with virus only. Next, we isolated the plant leaf DNA infiltrated with CRISPR reagents and virus infectious clones to check the Cas9 endonuclease activity to target virus DNA. Sequencing of PCR amplicons encompassing gRNA targets showed the indels induced by Cas9 endonuclease (Fig. 7).

## Discussion

Among plant viruses, begomoviruses are the major factors in crop losses every year and consequently threaten the global food security.^25^ Development of disease resistance crop varieties is the most sustainable approach to overcome viral diseases. Control of insect vectors with pesticides and removal of infected plants by manual inspection have been ineffective, costly and time consuming. Engineering plant immunity to impart plant virus resistance offers great potential for reducing crop losses, increasing plant productivity, and ensuring food security.^26^ The CRISPR-Cas9 system has been emerged as a simple, efficient, and precise genome engineering tool for the development of genetically improved crop plants. It has become a method of choice in genome editing experiments in plant research.^27,28^ Previously, CRISPR-Cas9 mediated virus resistance has been achieved in multiple plant species. The CRISPR-Cas9 based targeting of coding and non-coding virus DNA sequences with different efficiencies has been reported, demonstrating lowering of virus titer and delayed symptom appearance.^16–21^

In this study, we investigated the efficacy of CRISPR-Cas9 system for targeting overlapping genes of three most prevalent CLCuV strains in Pakistan. We assessed the virus accumulation and indels formation at three different sites of six overlapping genes of CLCuV. Next, we examined the additive effects of multiplexed virus gene targeting against multiple CLCuV strains. Moreover, we also checked that whether virus variants were generated after targeting with multiplex CRISPR-Cas9 cassette.

We tested efficiencies of three gRNAs targeting six overlapping genes of three most prevalent strains of CLCuV to interfere with virus genome. *N. benthamiana* leaves were infiltrated with three gRNA constructs followed by virus infectious clones [CLCuBuV, CLCuKoV, CLCuMuV/CLCuMuB). All infiltrated plants showed mild and delayed virus infection compared to control plants infiltrated with virus only. Virus accumulation in infiltrated leaves was quantified to further validate the results. The qPCR results demonstrated less virus accumulation in systemic leaves when compared with control plants as described in the results. Moreover, expression analysis of all three gRNAs and Cas9 in agroinfiltrated plants also confirmed the activity of the CRISPR-Cas9 constructs.

Viral DNA accumulation reduced in all co-infiltrated plants when compared to control plants. Up to 70% reduction in virus accumulation was recorded when overlapping sequences of CLCuVs genome were targeted. As,^22^ found emergence of virus variants by targeting two overlapping genes of African cassava mosaic virus (ACMV], and required resistance was not achieved possibly due to single site targeting and subsequently repaired by NHEJ pathway. Based on above results, it is inferred that targeting overlapping genes of virus could be an efficient system to suppress virus replication. But, co-delivery of multiple gRNA cassettes did not trigger any additive effect on the accumulation of the virus genome. It was reasoned that multiple gRNAs are not expressed in the same cell equally, thereby hindering simultaneous cleavage of virus genome.^16^ It was suggested that multiplexing several gRNAs in a single vector could potentially deliver its additive effect.^29^ Accordingly,^30^reported that, transgenic *N. benthamiana* plants expressing Cas9 and dual gRNAs that target different regions of the CLCuMuV genome confer complete resistance to virus infection, thereby demonstrating a novel approach for engineering resistance to geminiviruses. Therefore, to test inhibition virus proliferation after simultaneously targeting of all genes of CLCuV DNA-A, three efficient gRNAs causing low virus accumulation by qPCR were multiplexed in a single vector.

The ability of multiplex CRISPR-Cas9 cassette to inhibit CLCuV genome was tested in *N. benthamiana* plants via agroinfiltration. Virus titer analysis revealed that virus accumulation was reduced significantly when we targeted virus DNA at multiple sites as compared to single gRNA target. Next, we tested the ability of stable multiplex cassette expressing *N. benthamiana* line to interfere with CLCuVs. A multiplex cassette was introduced into *N. benthamiana* by using Agrobacterium mediated leaf disc transformation method. Transgenic *N. benthamiana* line, expressing high level of Cas9 and gRNAs along with control (non-transgenic plants) was selected for infiltration with the virus infectious clones. Severe virus symptoms were recorded on the control non-transgenic plants, whereas very few virus symptoms were appeared on transgenic plants as compared to individual gRNAs, indicating higher efficacy of CRISPR-Cas9 multiplex vector to thwart virus infection. The qPCR with DNA extracted from both transgenic and non-transgenic plants further confirmed the efficiency of multiplex vector to inhibit CLCuVs proliferation in these plants. Moreover, DSB detection by T7EI assay also clearly demonstrated the ability of the CRISPR-Cas9 system to target CLCuVs genome. Silencing of the integrated gene cassette is common in crop plants, particularly when plants are engineered for virus resistance.^31,32^ Therefore, we also tested CRISPR-Cas9 multiplex cassette to thwart virus infection in T1 and T2 generations. Results indicated the consistency and inheritance of multiplex cassette against virus interference. Finally, we also tested multiplex CRISPR-Cas9 cassette in cotton plants to interfere CLCuV infection. Virus titer determined by qPCR was found less in multiplex infiltrated cotton plants as compared to plants infiltrated with virus only. Moreover, sequencing results also confirmed the activity of Cas9 endonuclease in cotton plants.

This study provides insight into the potential use of CRISPR-Cas9 system for establishing broad-spectrum and durable virus resistance in plants. Since, error prone NHEJ repair of the targeted regions of different Geminiviruses is very fast,^17^ targeting single region may lead to the emergence of mutated virus molecules capable of replication, proliferation, and systemic infection. Notably, ability of virus genome to replicate and escape Cas9 interference was earlier observed in ACMV, TYLCV, and HIV-1 viruses.^19,22,32^ Moreover, it is likely that targeting of viral DNA with multiple gRNAs would result long deletions rendering virus incapable of replication and systemic infection.^17^ Therefore, it would be a plausible strategy to achieve effective interference in which CRISPR-Cas9 system concurrently mutates several essential parts of virus.^30^ However, the role of targeting specific viral DNA sequences to promote recombination of virus itself under field conditions, is still to be determined.

## Conclusion

The short genome of begomoviruses contains specific DNA sequences necessary for viral replication, host infection and interacting with host defense systems. For targeted viral DNA interference, focus should be on the sequences that can provide efficient and better interference. At the same time, targeting multiple regions of the virus genome would greatly facilitate to overcome virus ability to recover from DNA repair. These chopped viral DNA fragments would ultimately be degraded. In conclusion, multiplex gRNA approach would be considered for developing durable and broad-spectrum resistance against plant viral diseases.
